# An Epidemiologic Transition of Cardiovascular Disease Risk in Carriacou and Petite Martinique, Grenada: the Grenada Heart Project, 2005-2007

**DOI:** 10.5888/pcd9.110167

**Published:** 2012-04-19

**Authors:** Robert C. Block, Ann M. Dozier, Leslie Hazel-Fernandez, Joseph J. Guido, Thomas A. Pearson

**Affiliations:** Department of Community and Preventive Medicine, University of Rochester; University of Rochester School of Medicine and Dentistry, Rochester, New York; Competitive Health Analytics, Inc, Miami, Florida; University of Rochester School of Medicine and Dentistry, Rochester, New York; University of Rochester School of Medicine and Dentistry, Rochester, New York

## Abstract

**Introduction:**

The epidemiologic transition has made chronic disease a major health threat in the Caribbean and throughout the world. Our objective was to examine the pattern of lifestyle factors associated with cardiovascular disease (CVD) in Grenada and to determine whether the prevalence of CVD  risk factors differs by subgroups.

**Methods:**

We conducted a cross-sectional study of adult Grenadians between 2005 and 2007. We used a population-wide, community-based approach by adapting the World Health Organization's STEPwise Approach to the Surveillance of Chronic Disease survey for a local context. We collected behavioral, anthropometric, and blood sample data to assess the prevalence of CVD risk factors.

**Results:**

An estimated 64% (n = 2,017) of 3,167 eligible adults participated in our study (60% women). With increasing age, consumption of fried foods declined, whereas fish intake increased. Adults aged 45 to 54 years had the highest obesity rate (39%). Large waist circumference was more common among women than among men. According to National Cholesterol Education Program criteria, 29% of participants had metabolic syndrome (47% ≥65 y; 36% women vs 17% men). Approximately one-fifth of participants had lived outside Grenada for more than 10 years. Participants who had migrated tended to be older and have different CVD risk factors than those who had never migrated.

**Conclusion:**

In the midst of an epidemiologic transition in the Caribbean nation of Grenada in which CVD risk is increasing, dietary risk factors are most prevalent among women and among all adults younger than 55.

## Introduction

The epidemiologic transition (1,2) is the shift in mortality from childhood infectious diseases, nutrient deficiencies, and epidemics at all ages to degenerative and lifestyle-related diseases at a later age. Many developing countries are undergoing this transition. Improved public health measures and medical care help people live longer, more productive lives (1); concurrently, these countries' populations often experience changes in diet and reductions in physical activity that lead to higher prevalence of cardiovascular disease (CVD) and CVD risk factors (3).

Low- and moderate-income nations account for approximately 78% of the world's deaths from noncommunicable disease and 85% of noncommunicable disease prevalence (3). Noncommunicable disease occurs disproportionately among people in their most productive years of youth and middle age (4). A dramatic shift is expected to occur by 2025 when noncommunicable diseases will account for an increasing proportion of disease burden in low-income nations. Because of rapid industrialization and genetic factors, the health effects of Westernization have received much attention, primarily in South Asia and other large Asian countries and in Latin America. Less attention has been paid to the Caribbean region, despite its diverse and generally less affluent populations.

Grenada, a developing nation in the southern Caribbean, lies north of Trinidad and Tobago and is part of the chain of islands called the Grenadines (2). The nation has a population of approximately 95,500: 90,000 on Grenada, 4,600 on Carriacou, and 900 on Petite Martinque. Inhabitants are primarily of African descent. In 2005, the World Heart Federation sponsored the Grenada Heart Project (GHP) to examine effects of the epidemiologic transition on CVD risk in the nation (2). In selecting this Western Hemisphere country, the WHF considered several factors. First, Grenada's geographic isolation made it amenable to public health measures focusing on the modification of local health practices, which are highly influenced by community health leaders. These leaders could play a more active role in the dissemination of interventions than those in other countries. Second, Grenada was early in the epidemiologic transition, a stage at which interventions that mitigate the transition's negative health consequences are more likely to work. Third, its population of approximately 95,000 was a manageable number for the project.

Our objective was to study the epidemiologic transition and CVD risk factors in the islands of Carriacou and Petite Martinique in Grenada, which has low rates of coronary heart disease, as does most of the Caribbean (5). We also investigated whether the epidemiologic transition in these islands is accelerated in subgroups of the population, particularly among Grenadians who have migrated (lived outside of Grenada) for some portion of their lives.

## Methods

### Population and study design

Our study was cross-sectional. We conducted the study in the Grenadian islands of Carriacou and Petite Martinique between 2005 and 2007. It was approved by a community advisory board composed of local University of Rochester and World Heart Federation stakeholders and the institutional review boards representing the University of Rochester and St. George's University School of Medicine in Grenada.

We used community-based, participatory research strategies as part of GHP to estimate the prevalence of CVD and its risk factors in Grenada. GHP was conducted in 3 phases: 1) community engagement, 2) a rapid assessment process of cultural and societal characteristics, and 3) the quantitative data collection phase. Community engagement was an essential component of GHP. Thus, the smaller islands of Petite Martinique and Carriacou were well suited to our study because of their small, manageable populations. We partnered with key national health organizations and worked directly with a local advisory board representing both islands. We implemented rapid assessment process procedures to assess local knowledge, societal attitudes, and individual behaviors in regard to CVD. This process and our findings from this first phase were previously reported (2). The study described here, the quantitative portion of the GHP, was a secondary analysis of these data.

### Recruitment

We recruited participants through a public education campaign, including local radio station announcements and door-to-door visits by local study staff, in conjunction with the Grenada Ministry of Health. Local study staff scheduled meetings with potential survey participants; however, we provided for walk-ins and in-home administration of the survey. Our data collection sites were located in public health service clinics staffed by local clinic medical personnel and trained local study staff. All adults aged 18 or older were eligible. We obtained written informed consent before participation, provided postparticipation counseling, and referred participants to their doctors if necessary. We used trained US and local study staff to administer surveys and obtain biological measurements.

### Data collection

During this second phase of the GHP, we conducted a survey of lifestyle and CVD risk factors. On the basis of the qualitative study results from GHP's first phase (2), we expanded and adapted the World Health Organization's STEPwise Approach to Chronic Disease Risk Factor Surveillance survey (6) to align the survey and methods with local attitudes and customs. We added questions about migration to countries outside of Grenada; consumption of fish, poultry, red meat, and fried and high-carbohydrate foods; access to a vegetable garden; and family or personal history of heart disease and stroke. Because we added some questions about diet to the survey after its use on Petite Martinique, data from those questions are reported only for Carriacou. This interviewer-administered survey took approximately 10 to 15 minutes to administer. We measured height and weight with a combined stadiometer and balance beam scale (SR160, SR Scales, Tonawanda, New York). Blood pressure was recorded 3 times using an OMRON automatic blood pressure device (OMRON Healthcare Inc, Bannockburn, Illinois) with an appropriate cuff size. Because  the local culture regarded phlebotomy negatively, we used a finger-stick method for blood chemistry analyses. For most participants, we completed all components of the testing during 1 visit. Physician investigators counseled participants about all health issues revealed by their study data.

### Laboratory methods

Participants fasted for 12 hours before blood collection. For cholesterol testing, we used Cholestech LDX analyzers (Medical Technology Resources, Salt Lake City, Utah), which employ a finger-stick method that has been validated against standard lipid measurement with phlebotomy (7). The LDX system measures mg/dL of total cholesterol, high-density lipoprotein (HDL) cholesterol, low-density lipoprotein (LDL) cholesterol, and triglycerides and calculates the ratio of total to HDL cholesterol ratio and LDL cholesterol in mg/dL. If values of 1 or more of the following measurements were classified by the LDX as "not applicable" (correlating with a total cholesterol <50 mg/dL or >300 mg/dL and blood glucose <100 mg/dL or >300 mg/dL) or a counselor or supervisor requested repeat testing because of inadequate blood volume or defective testing materials, we repeated testing and used the average of the 2 values.

### Variables

We defined metabolic syndrome according to the National Heart, Lung, and Blood Institute and the American Heart Association guidelines as the presence of 3 or more of the following: waist circumference greater than 102 cm in men and 88 cm in women; triglycerides of at least 150 mg/dL; HDL cholesterol lower than 40 mg/dL in men and 50 mg/dL in women; blood pressure of at least 130/85 mm Hg; or fasting blood glucose at or higher than 110 mg/dL (8). We used standard methods to calculate body mass index (BMI).

### Statistical analyses

We used univariate and bivariate descriptive statistics to examine the distribution of key behavioral and biological factor variables across sex and age groups. We used the Cochran-Armitage trend test (categorical variables), the Kruskal-Wallis test (ordinal variables), and Spearman correlation (continuous variables) to test for trends. We tested for significant differences between more than 2 continuous variables using ANOVA and used χ^2^ analyses to assess the independent relationships of categorical variables. Significance was defined by a 2-sided *P* value of less than .05. Data analyses were conducted using SAS/STAT software, version 9 (SAS Institute, Inc, Cary, North Carolina).

We used multivariate regression to determine whether the presence of well-established Framingham Heart Disease CVD risk factors (9) differed significantly between the 2 migration groups and thus may have been associated with the health behaviors among people in these different groups. We used 2 models, 1 including physical activity and 1 excluding physical activity (which is not a Framingham risk factor).

## Results

An estimated 64% (n = 2,017) of 3,167 eligible adults from Carriacou and Petite Martinique aged 18 to 104 participated in our study, 60% of them female. The mean age of participants was 47; 79% were black, and approximately half had only a primary school education (Table 1). Most participants were native Grenadian residents of Carriacou; 72% had lived abroad at some point in their lives, and 21% had lived abroad for at least 10 years. Rates of self-reported  hypertension, diabetes, and heart disease were 30%, 13%, and 6%, respectively, and were significantly higher among women than men.

Study participants in younger age groups tended to have less healthy diets (ie, types of food eaten and frequency) than their older counterparts (Table 2). Younger participants ate more fried meat, chicken, and duck and other types of fried food than their older counterparts. Younger women ate less fish than older women. Reported fruit and vegetable consumption did not differ by age for either sex, although garden access increased with age among women. Younger men and women were more likely to walk or bike than their older counterparts, and younger men were less likely to be sedentary than older men.

Many health indicators worsened with age for both men and women (Table 3); these indicators included systolic blood pressure, hypertension, diabetes, and heart disease. The trends of worsening health indicators were more pronounced among women than men. Smoking rates were 20% to 31% for men; women had much lower rates, 3.6% to 10.0% (data not shown). The relationships between age and hypertension, diabetes, and heart disease were consistent when stratifying by BMI for men and women combined (P < .001). The proportions of those we categorized as normal weight, overweight, and obese were approximately evenly divided (610-675 per category). Nearly all (85%) obese participants older than 65 had 1 or more diagnoses of hypertension, diabetes, and heart disease; proportions were nearly identical among men and women (84% vs 85%) compared with only 21% of participants younger than 35. Overall, 42% of women and 17% of men were obese. Participants aged 45 to 54 had the highest rate of obesity, 39%; 33% were overweight, and 28% were normal weight. More women in this age group were obese (51% vs 25% of men), and women had larger waist circumferences than men for each age group. The prevalence of metabolic syndrome was 29% for all adults, and rates were highest in those aged 65 or older (47%). The rate among all women was 36% versus 17% for men (P < .001) and among women aged 65 or older was 60% for women versus 23% for men (P < .001).

We found differences specific to migration between those who had migrated to other countries (dichotomized into ≤10 y and >10 y) and those who had not lived outside Grenada (Table 4). Women who had resided outside Grenada for more than 10 years were less likely to consume fried foods and more likely to eat fruit and vegetables than women who had lived outside the country for 10 years or less. Men who had lived outside Grenada for more than 10 years were more likely to drink alcohol and be sedentary than men who had lived outside Grenada for 10 years or less. Both men and women with longer periods of migration were more likely to be older and to have more CVD risk factors (hypertension, diabetes, and elevated blood glucose) than those who had not lived outside Grenada. Those who had lived outside Grenada for more than 10 years were significantly older than those who had lived outside Grenada for 10 years or less (*P* < .05). Thus, age may have influenced lifestyle and cardiovascular disease risk factors. Tobacco use, sex, lipoproteins, the presence of diabetes or hypertension, and activity levels were not significantly different.

## Discussion

We found that the epidemiologic transition from communicable to noncommunicable diseases is well underway in the 2 small, rural Grenadian islands of Carriacou and Petite Martinique. Despite the lack of fast-food restaurants and a reported traditional diet of healthy fresh produce and seafood, about one-third of study participants were obese, and almost one-third had metabolic syndrome. Although the epidemiologic transition may be a recent phenomenon in the southern Caribbean region and although these 2 islands are rural and geographically isolated, CVD risk factors are at least as common among residents of Carriacou and Petite Martinique as they are in African Americans (10,11). The difference in diet among age groups indicates potential future public health problems associated with CVD risk. A dramatic increase in CVD in the next 10 to 20 years is expected, particularly given the dietary patterns among younger Grenadians, who are consuming less fish and more red meat, poultry, fried meats, and other fried foods than their older counterparts. Additionally, these dietary patterns are more pronounced among women than among men. Among Grenadians, having lived abroad is actually associated with a healthier diet and may be a modifying factor in this epidemiologic transition, although this may depend on a person's age.

The transition to CVD risk factors is occurring in Grenada in conjunction with a tradition of very high fish consumption, which is higher among Grenadians of all ages than in the United States (12). The consumption of fish and its constituent omega-3 fatty acids, eicosapentaenoic acid, and docosahexaenoic acid, has been consistently associated with improved cardiovascular health (13). Beneficial effects of these unique fatty acids include a reduction in risk for sudden cardiac death (14-16), nonfatal acute coronary syndromes (12,17), and hospital admissions among people with congestive heart failure (18). In addition, the replacement of saturated fat with such long-chain polyunsaturated fatty acids has been associated with reduced levels of insulin resistance (19-21), the current most widely accepted risk factor for metabolic syndrome (22). Moreover, even in Japan, whose residents have a much higher level of fish consumption than those of most other countries, eicosapentaenoic acid supplementation has been associated with reduced risk of nonfatal coronary events (23). These facts may help explain the lower prevalence of CVD among Grenadians than would be expected, based on their risk factors.

Abdominal obesity and associated lifestyle factors are more prevalent among women than men in Grenada. Abdominal obesity and metabolic syndrome are more prevalent among women than among men in other developing countries and are associated with a sedentary lifestyle (24-27). Another island with a population of African origin with very high levels of fish consumption is the Republic of Seychelles. The Seychelles is experiencing rapid economic development, and obesity is common; obesity rates in the Seychelles (30%) are similar to those in Grenada (25%) (28). The age-standardized rate of obesity in adults in the Seychelles is 5 times greater among women than men (20.9 vs 4.2%) (29). Studies report that women in the Seychelles are less physically active than men, as is the case in the non-African populations of urban east India (30) and Saudi Arabia (31). Although obesity issues among women are not confined to those of African ethnicity, studies of African Americans, particularly women, indicate a lack of social pressure to be slim and a reduced stigma associated with obesity (32,33).

Emigration to Western countries has been associated with increased risk for CVD because of many factors, including transition to more urban environments, physical inactivity, a more energy-rich and atherogenic diet (3), gene-environment interactions, stress, and ethnic susceptibility (34). More sedentary lifestyles and high-fat diets have been associated with urbanization and its associated affluence and disruptions in traditional cultures. Another factor associated with urbanization is a transition to work that is devoid of physical labor. In developing rural countries, underprivileged and increasingly urban residents are predominantly lean, are engaged in physical labor, and have a very low risk of CVD. The trend in Grenada, however, is that people who have lived outside that country for more than 10 years tend to have healthier diets than those who have not lived outside Grenada or have done so for 10 years or less. This trend is particularly striking among women, who already have more worrisome dietary trends than men. Thus, migration status and fish consumption among Grenadians may be important considerations in preventing a more rapid epidemiologic transition in this poor, developing nation of ethnically African inhabitants. These trends, which may influence the health of future generations of Grenadians and residents of other Caribbean nations, warrant further study.

Our study had several strengths and limitations. Involvement of local community members on the research team enhanced trust between the community and the US investigators. The study sample was recruited using a community-based participatory research strategy that leveraged community leaders and community-based and community-driven marketing techniques. A limitation is that participants represented a self-selected convenience sample. Although this sampling technique is not optimal, we were able to recruit an estimated 57% of the residents of the islands of Petite Martinique and Carriacou. Much of the data collected were self-reported. This was a cross-sectional study; therefore, an assessment of any causal relationship of lifestyle and migration factors with CVD is not possible. Our ability to gather highly detailed dietary and other lifestyle data was limited by researchers' concerns that a long questionnaire would limit the ability to recruit an adequate study sample and local community concerns about medical research.

Grenada, a developing nation that is experiencing an epidemiologic transition from infectious to chronic, degenerative diseases, has substantial rates of CVD risk factors among young and middle-aged adults. Because dietary risk factors are more prevalent among young women than young men, public health measures to reduce the prevalence of CVD in Grenada may be most effective if they target women before they reach adulthood. Although migration from developing to developed nations is commonly associated with unhealthy lifestyle changes, among Grenadians, living in a Western country for more than 10 years is associated with better dietary habits than among those who have not lived outside Grenada. Further study of the relationship of migration to CVD risk in people living in the Caribbean is necessary to understand the process of epidemiologic transition and to stem the increase of chronic diseases in countries with challenged medical care systems.

## Figures and Tables

**Table 1.** Characteristics of Participants (N = 2,017) in a Study of the Epidemiologic Transition of Cardiovascular Disease Risk in Residents of Carriacou and Petite Martinique, Grenada, 2005-2007^a^

Characteristic	Total	Men (n = 807 )	Women (n = 1,210)	*P* Value^b^
Age, mean (SD), y	47.3 (18.0)	46.4 (17)	48.0 (19)	.05
Black race	1,594 (79)	624 (77)	970 (80)	.50
Attended primary school only (aged 5-11 y)	1,013 (51)	396 (49)	617 (51)	.60
Income (ECD) ≥$1500/year	330 (38)	169 (21)	161 (13)	<.001
Resides on Carriacou	1,791 (89)	704 (87)	1,087 (90)	.61
Born in Grenada	1,803 (90)	713 (88)	1,090 (90)	.94
Has lived outside Grenada	1,443 (72)	607 (75)	836 (69)	<.001
Lived outside Grenada <10 y vs not lived outside	1,024 (51)	436 (54)	588 (49)	<.001
Lived outside Grenada >10 y vs not lived outside	419 (21)	171 (21)	248 (20)	.02
Hypertension	596 (30)	171 (21)	425 (35)	<.001
Diabetes	254 (13)	54 (7)	200 (17)	<.001
Heart disease	113 (6)	28 (4)	85 (7)	<.001

Abbreviations: SD, standard deviation; ECD, Eastern Caribbean dollars.

a Data were obtained from a questionnaire. Values are expressed as n (%), except for age.

b Men vs women, *t* test for age and χ^2^ test for all other variables.

**Table 2.** Dietary and Lifestyle Factors by Age and Sex in Carriacou and Petite Martinique, Grenada, 2005-2007^a^

Characteristic	Age, y					*P* Value^b^

**Men, Carriacou and Petite Martinique**	<35 (n = 233)	35-44 (n = 146)	45-54 (n = 172)	55-64 (n = 91)	≥65 (n = 144)	NA
Eats fish ≥twice/wk, %	78.1	84.9	83.7	81.3	80.6	.63
Eats fried meat/fish, %	80.3	70.1	77.8	68.5	61.4	<.001
No. of fruit servings/wk, median (IQR)	6.0 (10.0)	6.0 (11.0)	6.0(9.0)	6.0 (5.0)	6.0 (7.0)	.74
No. of vegetable servings/wk, median (IQR)	4.0 (5.0)	4.0 (5.0)	4.0 (5.0)	4.0 (5.0)	4.0 (5.0)	.91
Current smoker, %	20.3	24.1	31	25.3	22.1	.44
Drinks alcohol^c^, %	7.7	10.4	11.1	8.6	13.8	1
Walks or bikes continuously >10 min/d, %	78.3	69.2	74.6	71.3	65.3	.02
Leisure time spent sedentary >10 min/d, %	72.8	80.9	79.7	80.7	93.7	<.001

**Men, Carriacou only**	**n = 192**	**n = 126**	**n = 145**	**n = 75**	**n = 124**	**NA**

Eats red meat ≥twice/wk, %	22.9	18.3	16.6	16	14.5	.47
Eats chicken/duck ≥twice/wk, %	76.2	66.7	61.4	65.4	61.4	.004
Eats fried foods other than fried meat/fish ≥twice/wk, %	28.5	15	18.4	13.3	3.6	<.001
Eats carbohydrate-rich foods ≥twice/wk, %	63.4	65.6	59.6	55.1	58.8	.17
Has access to a vegetable garden, %	57.8	57	60.8	73.1	56.1	.53

**Women, Carriacou and Petite Martinique**	**n = 343**	**n = 222**	**n = 198**	**n = 155**	**n = 271**	**NA**

Eats fish ≥twice/wk, %	65.3	74.3	76.8	83.2	85.6	<.001
Eats fried meat/fish, %	80.8	73.5	65.5	56.7	50.9	<.001
No. of fruit servings/wk, median (IQR)	6.0 (11.0)	5.0 (7.0)	7.0 (11.0)	7.0 (11.0)	6.0 (11.0)	.67
No. of vegetable servings/wk, median (IQR)	4.0 (5.0)	4.0 (5.0)	4.0 (5.0)	4.0 (7.0)	4.0 (5.0)	.20
Current smoker, %	4	3.6	10	4.5	8.4	.02
Drinks alcohol^c^, %	3.2	4.9	6.5	7.6	2.2	0.93
Walks or bikes continuously >10 min/d, %	79.5	81.1	80.1	76.1	61.7	<.001
Leisure time spent sedentary >10 min/d, %	78.1	79.9	82.9	83.8	83.5	.53

**Women, Carriacou only^a^**	**n = 294^a^**	**n = 181**	**n = 165**	**n = 126**	**n = 237**	**NA**

Eats red meat ≥twice/wk, %	20.4	9.9	13.9	11.9	10.1	.003
Eats chicken/duck ≥twice/wk, %	20.4	9.9	13.9	11.9	10.1	.003
Eats fried foods other than fried meat/fish ≥twice/wk, %	82.8	69.7	71.4	53.7	57.4	<.001
Eats carbohydrate-rich foods ≥twice/wk, %	24.8	13.7	10.9	6	5.2	<.001
Has access to a vegetable garden, %	55.7	56	49.1	53.8	49.2	.11

Abbreviations: IQR, interquartile range; NA, not applicable.

a Data on some variables were available only for residents of Carriacou. Some data were collected only in Carriacou as they were added to this phase of the study, which occurred after information was first obtained from study completion in Petite Martinique

b *P* values for trend for variables calculated using the Spearman correlation coefficient. *P* values for trend for categorical variables calculated using the Cochran-Armitage trend test.

c Alcohol consumption defined as 1-2 drinks/d or 1-7 d/wk.

**Table 3.** Anthropometric Characteristics and Clinical Diagnoses by Age and Sex, Carriacou and Petite Martinique, Grenada, 2005-2007

Characteristic/Diagnosis	Age, y					*P* Value^a^

Men	<35 (n = 234)	35-44 (n = 142)	45-54 (n = 166)	55-64 (n = 89)	≥65 (n = 138)	NA
Systolic blood pressure, mean (SD), mm Hg	121.1 (11.9)	122.3 (13.2)	128.5 (17.3)	136.1 (21.0)	138.4 (21.5)	<.001
Diastolic blood pressure, mean (SD), mm Hg	69.2 (10.2)	72.1 (11.1)	77.7 (12.3)	78.3 (11.2)	75.5 (12.0)	<.001
Body mass index, kg/m^2^	25.4	27.2	26.6	26.6	25.8	.05
Waist circumference, in	33.3	36.0	35.7	36.4	36.3	<.001
Total cholesterol, mean (SD), mg/dL	171 (41)	194 (52)	193 (45)	195 (40)	185 (37)	<.001
LDL cholesterol, mean (SD), mg/dL	113 (38)	126 (48)	130 (41)	131 (38)	119 (35)	<.009
HDL cholesterol, mean (SD), mg/dL	43 (14)	47 (18)	48 (18)	45 (13)	49 (16)	<.004
Triglycerides, median (IQR), mg/dL	70 (50)	88 (72)	86 (63)	91 (62)	77 (42)	.08
Glucose, median (IQR), mg/dL	83 (11)	86 (16)	90 (18)	92 (19)	93 (17)	<.001
Hypertension, %^b^	14	21	38	51	60	<.001
Diabetes, %	1	4	12	16	20	<.001
Heart disease, %	1	1	2	4	12	<.001

**Women**	**n = 339**	**n = 209**	**n = 187**	**n = 148**	**n = 267**	

Systolic blood pressure mean (SD), mm Hg	107.2 (1.3)	117.0 (17.1)	127.9 (19.8)	135.5 (24.0)	147.6 (24.0)	<.001
Diastolic blood pressure, mean (SD), mm Hg	66.8 (9.8)	74.1 (11.9)	80.1 (13.5)	78.2 (13.1)	77.2 (12.1)	<.001
Body mass index, kg/m^2^	27.7	29.9	30.7	29.8	28.9	<.001
Waist circumference, in	34.3	36.5	36.8	37.7	37.3	<.001
Total cholesterol, mean (SD), mg/dL	165 (34)	178 (34)	188 (36)	213 (40)	203 (43)	<.001
LDL cholesterol, mean (SD), mg/dL	105 (32)	114 (33)	120 (34)	139 (41)	105 (32)	<.001
HDL cholesterol, mean (SD), mg/dL	46 (11)	45 (13)	48 (15)	49 (14)	50 (14)	<.001
Triglycerides, median (IQR), mg/dL	70 (44)	90 (66)	98 (64)	100 (58)	92 (65)	<.001
Glucose, median (IQR), mg/dL	80 (11)	84 (14)	88 (20)	94 (23)	96 (27)	<.001
Hypertension^b^, %	11	26	48	62	81	<.001
Diabetes^c^, %	3	11	15	30	37	<.001
Heart disease, %	2	2	4.1	11	18	<.001

Abbreviations: NA, not applicable; SD, standard deviation; LDL, low density lipoprotein; HDL, high density lipoprotein; IQR, interquartile range.

a *P* values for trend for variables calculated using the Spearman correlation coefficient. *P* values for trend for categorical variables calculated using the Cochran-Armitage trend test.

b A diagnosis of hypertension was based on participant self-report of hypertension or a blood pressure of 140/90 mm Hg or higher.

c A diagnosis of diabetes was based on participant self-report or fasting glucose of 140 mg/dL.

**Table 4.** Differences in Lifestyle and Cardiovascular Disease Risk Factors by Age, Sex, and Migration Status in Carriacou and Petite Martinique, Grenada, 2005-2007

Characteristic/Lifestyle Factors	Migration Category							

	No Migration^a^		Migration ≤ 10 y^b^		Migration > 10 y^c^		*P* Value^d^	

	Men (n = 185)	Women (n = 370)	Men (n = 430)	Women (n = 579)	Men (n = 171)	Women (n = 248)	Men	Women
**Age, mean (SD), y**	38.1 (14.7)	40.2 (16.2)	43.7 (15.1)	45.7 (17.3)	61.4 (14.3)	64.2 (14.1)	<.001	<.001
**Lifestyle risk factors**								
Eats fish ≥twice/wk, %	84	71	82	78	78	79	.44	.05
Eats red meat ≥twice/wk, %	14.8	14.6	19	13.9	20.1	13.2	.38	.66
Eats chicken/duck ≥twice/wk, %	65.8	78.2	69.7	69.7	61	56.2	.13	<.001
Eats fried foods other than fried meat/fish ≥ twice/wk, %	22.6	22.3	19.9	12.7	6.5	4.5	<.001	<.001
Eats carbohydrate-rich foods ≥twice/wk, %	61	61	61.8	61.8	59.1	59.1	.61	.88
Eats fried meat/fish^e^, %	79	73	73	69	67	55	.05	<.001
No. fruit servings/wk	8	8.3	8.7	8.4	9.2	11.2	.01	<.001
No. vegetable servings/wk	6.4	6	5.4	6	5.2	7.6	.57	<.001
Current smoker, %	27.3	4.9	22.5	7.1	24.3	5.3	.84	.82
Drinks alcohol^f^, %	7.1	1.9	9.2	4.7	17.1	6.9	.001	.007
Has access to a vegetable garden, %	60.4	53.2	60.2	59.6	58.3	53.3	.67	.53
Walks or bikes continuously >10 min/day, %	72.8	76.8	73.7	78.8	69.8	65.9	.39	<.001
Leisure time spent sedentary >10 min/day, %	77.2	83.8	78.2	79	89.2	83.4	.001	.66
**Cardiovascular risk factors**								
LDL cholesterol, mg/dL	120	117	122	120	119	130	.76	<.001
HDL cholesterol, mg/dL	45	45	46	47	47	51	.08	<.001
Triglycerides, mg/dL	99	98	101	107	84	106	.11	.04
Glucose, mg/dL	90	95	94	95	98	103	.02	<.001
Hypertension^g^, (%)	28	32	30	38	46	67	<.001	<.001
Diabetes h (%)	6	13	8	16	13	29	.01	<.001

Abbreviation: SD, standard deviation; LDL, low-density lipoprotein; HDL, high-density lipoprotein.

a Never lived outside Grenada.

b Lived outside Grenada ≤10 y or less.

c Lived outside Grenada >10 y.

d *P* values for trend for variables calculated using the Spearman correlation coefficient. *P* values for trend for categorical variables calculated using the Cochran-Armitage trend test.

e Includes respondents who answered "sometimes," "often," "usually," or "always."

f Respondents who reported alcohol consumption, defined as 1-2 drinks/d, 1-7 drinks/wk.

g Blood pressure ≥140/90 mm Hg.

h Fasting glucose ≥140 mg/dL.
